# Upregulation of CRISP3 and its clinical values in adult sepsis: a comprehensive analysis based on microarrays and a two-retrospective-cohort study

**DOI:** 10.3389/fimmu.2024.1492538

**Published:** 2024-11-18

**Authors:** An-qiang Zhang, Da-lin Wen, Xin-xin Ma, Fei Zhang, Guo-sheng Chen, Kelimu Maimaiti, Gang Xu, Jian-xin Jiang, Hong-xiang Lu

**Affiliations:** ^1^ State Key Laboratory of Trauma, Burn and Combined Injury, Third Military Medical University, Chongqing, China; ^2^ Graduate School of Xinjiang Medical University, Urumuqi, China; ^3^ Department of Traumatic Orthopaedics, General Hospital of Xinjiang Military Region, Urumuqi, China

**Keywords:** CRISP3, sepsis, prediction, meta-analysis, biomarker

## Abstract

**Background:**

Current lines of evidence indicate that cysteine-rich secretory protein 3 (CRISP3) is an immunoregulatory factor. Nevertheless, no study has explored the relationships between the values of CRISP3 and sepsis.

**Methods:**

We conducted a comprehensive literature search and meta-analysis from the Gene Expression Omnibus (GEO) and ArrayExpress to determine the expression of CRISP3 in sepsis patients. Then, we explored whether plasma CRISP3 could serve as a potential biomarker to predict the risk of sepsis via two retrospective trauma cohorts. We evaluated the prediction power using the area under the curve (AUC).

**Results:**

A total of 23 datasets were recruited for the comprehensive meta-analysis, and the combined standardized mean difference (SMD) of CRISP3 was 0.90 (0.50–1.30) (p < 0.001), suggesting that CRISP3 was overexpressed in sepsis patients. Meanwhile, sepsis patients had higher CRISP3 concentrations than non-sepsis patients in 54 trauma patients (p < 0.001). Plasma CRISP3 on admission was significantly associated with the incidence of sepsis [OR = 1.004 (1.002–1.006), p < 0.001]. As a predictive biomarker, CRISP3 obtained a better AUC [0.811 (0.681–0.905)] than C-reactive protein (CRP) [0.605 (0.463–0.735)], procalcitonin (PCT) [0.554 (0.412–0.689)], and Sequential Organ Failure Assessment (SOFA) [0.754 (0.618–0.861)]. Additionally, the clinical relationships between plasma CRISP3 and sepsis were verified in another trauma cohort with 166 patients [OR = 1.002 (1.001–1.003), p < 0.001]. The AUC of CRISP3 was 0.772 (0.701–0.834), which was better than that of CRP [0.521 (0.442–0.599)] and PCT [0.531 (0.452–0.609)], but not SOFA [0.791 (0.717–0.853)].

**Conclusion:**

Our study indicated and validated that CRISP3 was highly expressed in sepsis. More importantly, CRISP3 may serve as a latent biomarker to predict the risk of sepsis.

## Introduction

Conceptually, sepsis is now a life-threatening organ dysfunction caused by the dysregulation of the body’s response against infection (1). Recent data illustrated that 48.9 million cases and 11 million sepsis-related deaths occurred worldwide in 2017, and the mortality rate accounted for 19.7% of all global deaths (2). Over the decades, an advanced understanding of the underlying mechanisms of sepsis has gradually promoted the prevention, diagnosis, and novel therapeutic approaches for sepsis (3, 4). However, those increasing improvements have raised additional issues that have to be dealt with, and sepsis remains a major challenge for basic and clinical research (5–7). Therefore, it is urgent to identify pathophysiological targets participating in sepsis to promote individualized therapy of sepsis patients.

Cysteine-rich secretory protein 3 (CRISP3), a member of the CRISP family, is widely distributed in human tissue, such as the pancreas, prostate, epididymis, colon, and bone marrow under normal physiological conditions (8, 9). Currently, increasing studies have demonstrated that CRISP3 may be a novel predictive marker and therapeutic target for various diseases. Lee et al. (10) reported that CRISP3 was upregulated in immune-damaged mice infected with the hepatitis C virus (HCV), and CRISP3 reduced the replication of HCV at the early stage of infection. Liao et al. (11) demonstrated that the expression of CRISP3 was higher in chronic pancreatic tissue compared to normal tissue, and CRISP3 was involved in the destruction and remodeling of chronic pancreatitis. Arup et al. (12) identified that CRISP3 was upregulated in severe dengue patients, and CRISP3 was considered a putative marker of severe dengue. Moreover, Kooistra et al. (13) indicated that CRISP3 was distinctly dysregulated in pulmonary fibrosis caused by severe COVID-19. Lin et al. (14) demonstrated that a cluster of five genes, including CRISP3, was manually determined as potent discriminators of neutrophil function upregulated in long COVID-19 populations. Furthermore, dysregulated CRISP3 was observed in cervical cancer (15), gallbladder cancer (16), and breast cancer (17). Previous studies have indicated that upregulation of CRISP3 could promote leukocyte-mediated migration, neutrophil activation, and degranulation (14). Furthermore, CRISP3 was found to be homologous to pathogen-resistant proteins induced by infection in plants, which further supported the concept that CRISP3 may be an immunoregulatory factor (18). Therefore, we speculated that CRISP3 may participate in the development of sepsis. Nonetheless, the expression and clinical values of CRISP3 in sepsis were unclear.

In the current study, we conducted a systematic exploration via public datasets from the ArrayExpress and Gene Expression Omnibus (GEO) databases to determine and verify the clinical significance of CRISP3 in sepsis patients. Then, we attempt to explore whether plasma CRISP3 could serve as a potential biomarker to predict the occurrence of sepsis via two retrospective cohorts.

## Materials and methods

### Data collection

The expression of CRISP3 in sepsis was obtained from the GEO and ArrayExpress database using the following keywords: “CRISP3” and “sepsis”. The samples were filtered in the form of blood. Inclusion criteria were as follows: 1) diagnosis and/or prognosis of patients with adult sepsis, 2) the sample size in each group was ≥3, and 3) the expression values of CRISP3 were available. When multiple platforms were displayed, each platform was determined as the independent dataset. The search period ranged from the inception of the databases to August 2024.

### Study population

Two center cohorts were included in our study. Trauma patients were recruited from the Chongqing Emergency Medical Center and the intensive care unit (ICU) at the Department of Trauma Surgery of Daping Hospital between January 2020 and January 2022. Patients admitted in our study met the following criteria: 1) severe trauma patients with an Injury Severity Score (ISS) ≥16 points, 2) age from 16 to 65 years, and 3) patients admitted to the hospital within 24 hours after injury (19). Trauma patients with tumors, with autoimmune diseases, who are pregnant, and with other preexisting chronic diseases were excluded. A total of 220 injury patients were enrolled for further study, including 54 patients from Chongqing Emergency Medical Center (validation cohort 1) and 166 patients from Daping Hospital (validation cohort 2). All patients were followed up during their hospitalization. Demographic characteristics and clinical information were obtained. Acute Physiology and Chronic Health Evaluation (APACHE) II scores and Sequential Organ Failure Assessment (SOFA) scores were measured to assess the severity of the disease and organ failures. Sepsis cases were determined based on the definition of Sepsis-3 (1). When patients were admitted, their whole blood samples were collected immediately within 24 hours. The samplings and experiments obtained approval from the Institutional Ethics Review Board of the Army Medical University (TMMU202072). This study obtained signed consent from the patients or guardians.

### Sample collection, processing, and detection of plasma CRISP3

Whole blood samples were obtained and processed according to the methods previously described (19). Briefly, blood was immediately collected upon admission to the hospital with an EDTA-coated tube. Then, samples were centrifuged immediately at 3,000 rpm for 5 minutes at 4°C. Subsequently, the plasma samples were separated and stored at −80°C for further measurement. The CRISP3 concentrations in plasma were detected using a commercially available enzyme-linked immunosorbent assay (ELISA) kit (Abbexa, Cambridge, UK) in all samples according to the manufacturer’s instructions.

### Statistical analysis

Categorical parameters were displayed as numbers and percentages, and the χ^2^ test was performed. Continuous data expressed as the mean ± SD were compared with Student’s *t*-test or Mann–Whitney *U*-test. The relationships between plasma CRISP3 and trauma sepsis were determined using a logistic regression model. The correlations among different variables were evaluated using Spearman’s coefficient. To evaluate the discrimination capability, the area under the curve (AUC) of the receiver operating characteristic (ROC) curve was used.

For the main meta-analysis, CRISP3 expression levels were extracted from eligible datasets, and the expression values were presented as mean (M) and SD. The differences in CRISP3 values in the two groups were displayed on forest plots with the standardized mean difference (SMD) and the 95% confidence interval (CI). Cochran’s Q and the *I* ^2^ statistic were performed to calculate heterogeneity. When p < 0.05 or *I* ^2^ > 50%, a random-effects model was used. If not, a fixed-effects model was employed. Meanwhile, Egger’s and Begg’s tests were employed to determine publication bias. The differences were significant at p < 0.05. All statistical analyses were performed via SPSS version 18.0 and Review Manager 5.4 software.

## Results

### Meta-analysis indicated that CRISP3 mRNA elevated in sepsis patients

After screening the abstract and full text, 29 datasets from the GEO and ArrayExpress databases were in accordance with the eligibility criteria (20–41). The main features and detailed characteristics of each dataset included in the meta-analysis are illustrated in **Supplementary Table S1** and **Supplementary Table S2**. Among these datasets, eight datasets contained information on both the diagnosis and prognosis of sepsis. All included datasets were published from 2007 to 2024, and the majority of datasets were performed in the USA (six datasets) and Australia (five datasets). The sample sizes were 8–802, and the major datasets (22, 75.86%) had more than 50 samples.

**Figure 1:**
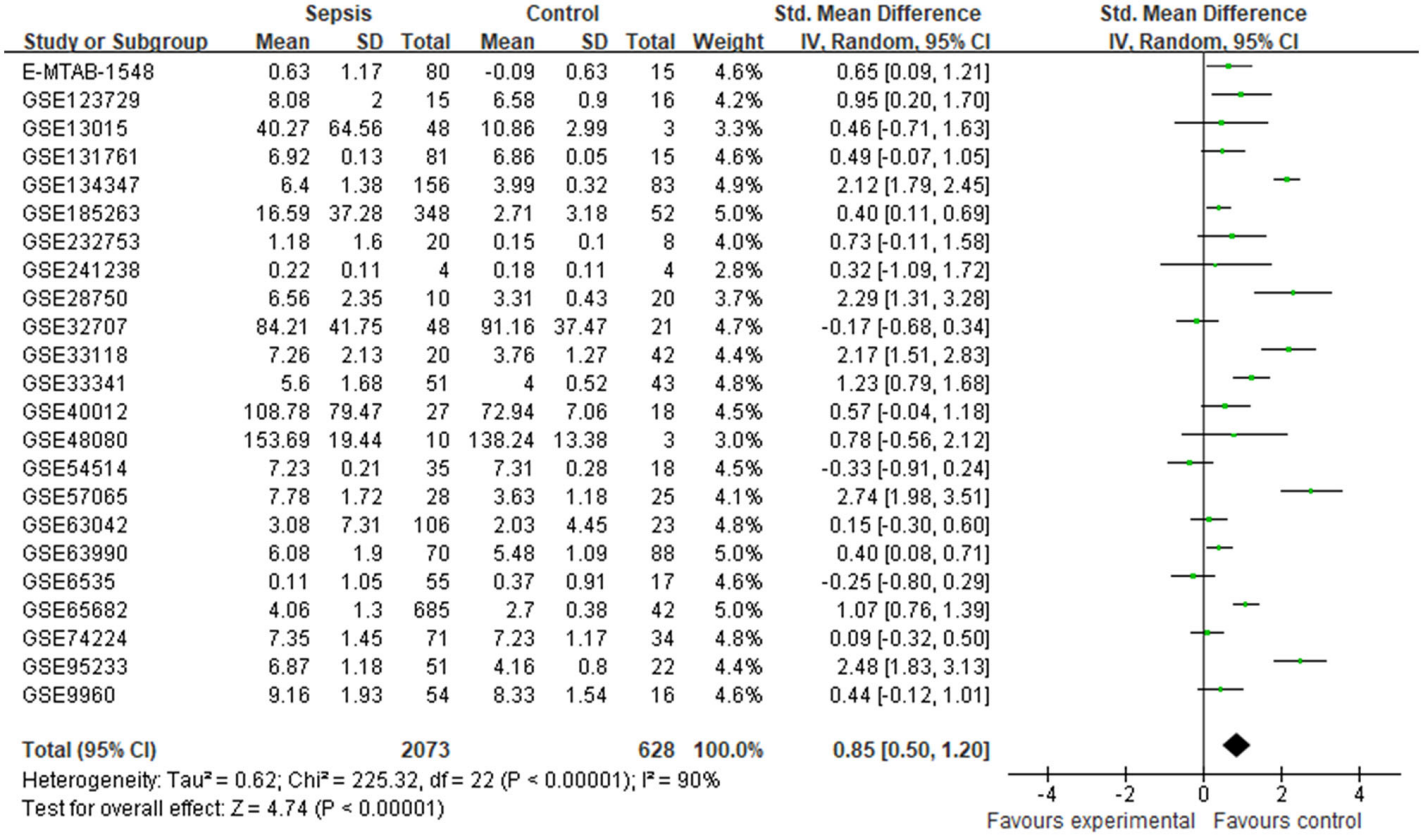
Meta-analysis of CRISP3 for the development of sepsis. Forest plot of CRISP3 expression in sepsis and controls. Sepsis, n = 2,073; controls, n = 628; SMD = 0.85 (0.50–1.20), p < 0.001; *I* ^2^ = 90%, p < 0.001. CRISP3, cysteine-rich secretory protein 3; SMD, standardized mean difference.

Finally, 23 datasets were considered to explore the expression levels of CRISP3 mRNA between controls and sepsis patients. The results indicated that a random-effects model was performed due to an apparent heterogeneity (p < 0.001, *I* ^2^ = 90%), and a remarkable overexpression of CRISP3 mRNA was observed in the sepsis patients (n = 2,073) compared with the controls (n = 628) [SMD = 0.85 (0.50–1.20), p < 0.001] (**Figure 1**). The asymmetrical funnel plot indicated obvious publication bias (**Supplementary Figure S2A**). In contrast, no significant difference in CRISP3 was detected between the sepsis survival and dead groups in the meta-analysis. As illustrated in **Supplementary Figure S1**, 14 datasets were used to explore the values of CRISP3 between the sepsis survival (n = 1,156) and dead patients (n = 777). The results demonstrated that no apparent heterogeneity was determined (p = 0.15, *I* ^2^ = 29%), and a fixed-effects model was applied. No remarkable upregulation of CRISP3 was found in the non-survival patients [SMD = −0.04 (−0.16 to 0.07), p = 0.46] (**Supplementary Figure S1**). No publication bias was observed via a metrical funnel plot (**Supplementary Figure S2B**). These results suggested that CRISP3 may not affect the prognosis of sepsis patients.

### Patient characteristics

To verify the plasma expression of CRISP3 in sepsis patients, two independent injury cohorts were recruited from different hospitals. Just as presented in **Table 1**, the majority of patients were male (75.93% and 73.49%). The patients were mostly young (mean age, 45.68 ± 13.23 and 45.24 ± 11.31). Most patients had severe injuries (mean ISS, 23.72 ± 9.28 and 27.09 ± 12.50). Sepsis morbidity rates were 40.74% and 32.53% in the validation cohort 1 and validation cohort 2, respectively. Gram-negative bacteria took up most of the pathogenic microorganisms (45.45% and 77.78%, respectively). Pneumonia, primary bloodstream, and secretion infections were the main infection sites. Furthermore, the SOFA scores at the initial 24 hours after injury were 1.93 ± 1.48 and 3.47 ± 1.80. The APACHE II scores at the initial 24 hours after trauma were 8.54 ± 4.57 and 10.12 ± 5.38.

**Table 1:** Basic characteristics of trauma patients.

Variables	Validation cohort 1 (n = 54)	Validation cohort 2 (n = 166)	p-Value*
Female/male, n (%)	13/41 (24.07/75.93%)	44/122 (26.51%/73.49%)	0.723
Age (years)	45.68 ± 13.23	45.24 ± 11.31	0.479
ISS	23.72 ± 9.28	27.09 ± 12.50	0.833
Glasgow Coma Scale (GCS) initial	13.69 ± 2.39	13.00 ± 3.46	0.188
APACHE II scores initial	8.54 ± 4.57	10.12 ± 5.38	<0.001
SOFA scores initial	1.93 ± 1.48	3.47 ± 1.80	0.638
Sepsis, n (%)	22 (40.74%)	54 (32.53%)	0.730
Pathogens, n (%)			
Gram-negative	10 (45.45%)	42 (77.78%)	
Gram-positive	4 (18.18%)	7 (12.96%)	0.009
Others	8 (36.36%)	5 (9.26%)	
Source of infection, n (%)			
Blood	4 (18.18%)	14 (25.93%)	
Sputum	7 (31.82%)	7 (12.96%)	
Urine	2 (9.09%)	14 (25.93%)	0.027
Secretions	4 (18.18%)	11 (20.37%)	
Others	5 (22.73%)	6 (11.11%)	
ICU days	2.98 ± 7.59	14.29 ± 15.18	0.206

ISS, Injury Severity Score; APACHE, Acute Physiology and Chronic Health Evaluation; SOFA, Sequential Organ Failure Assessment; ICU, intensive care unit.

*Categorical variables were compared using the χ^2^ test, and continuous variables were compared using ANOVA test.

### Plasma CRISP3 significantly increased in sepsis patients after trauma

**Figure 2:**
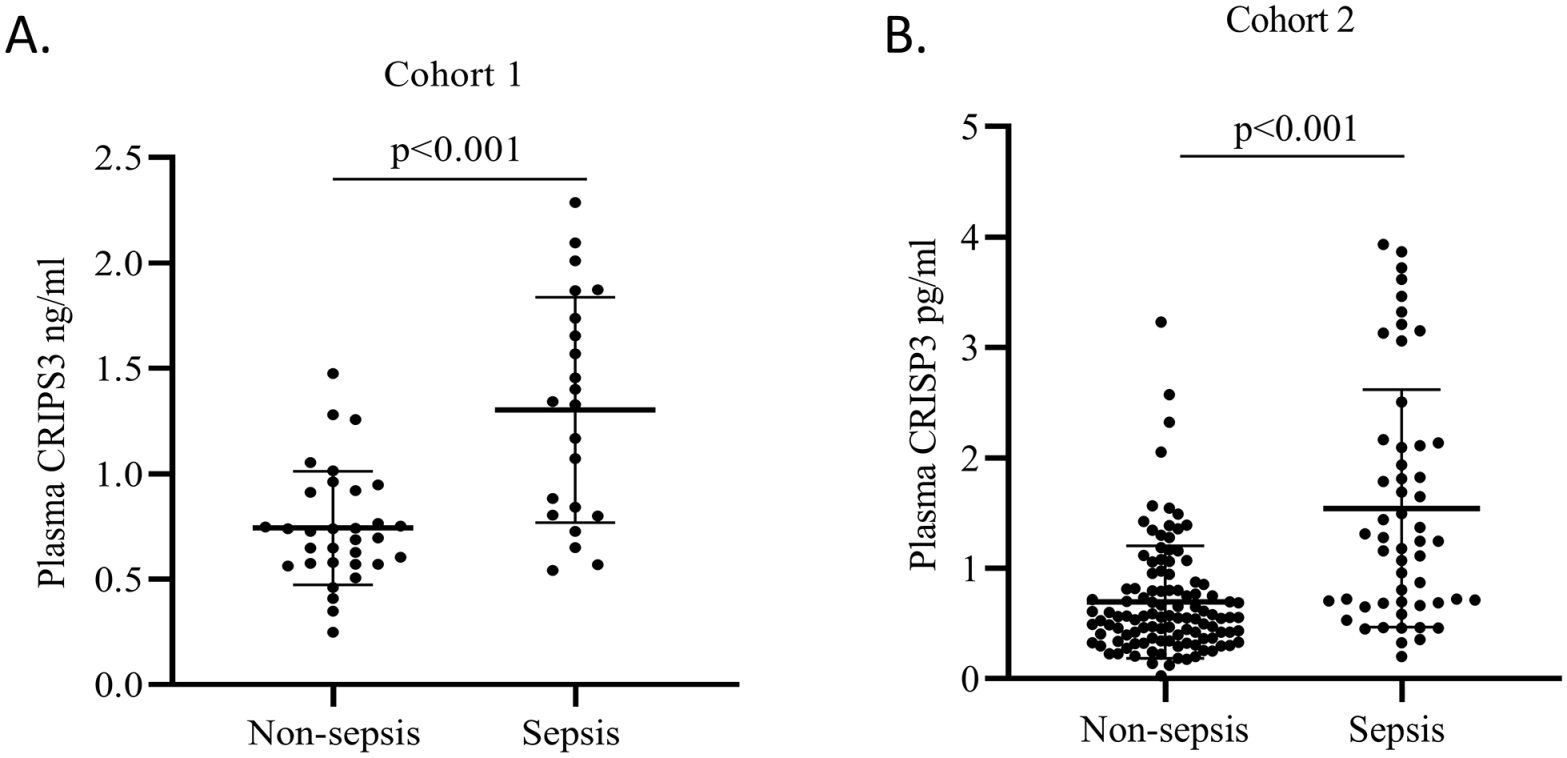
Plasma CRISP3 concentrations increased in the sepsis patients. **(A)** The sepsis patients had higher CRISP3 concentrations than non-sepsis patients in 54 trauma patients (1.305 ± 0.535 ng/mL vs. 0.743 ± 0.270 ng/mL p < 0.001). **(B)** The CRISP3 concentrations of sepsis elevated in sepsis patients compared with non-sepsis patients in 166 trauma patients (1.542 ± 1.075 ng/mL vs. 0.696 ± 0.509 ng/mL, p < 0.001). CRISP3, cysteine-rich secretory protein 3.

**Figure 3:**
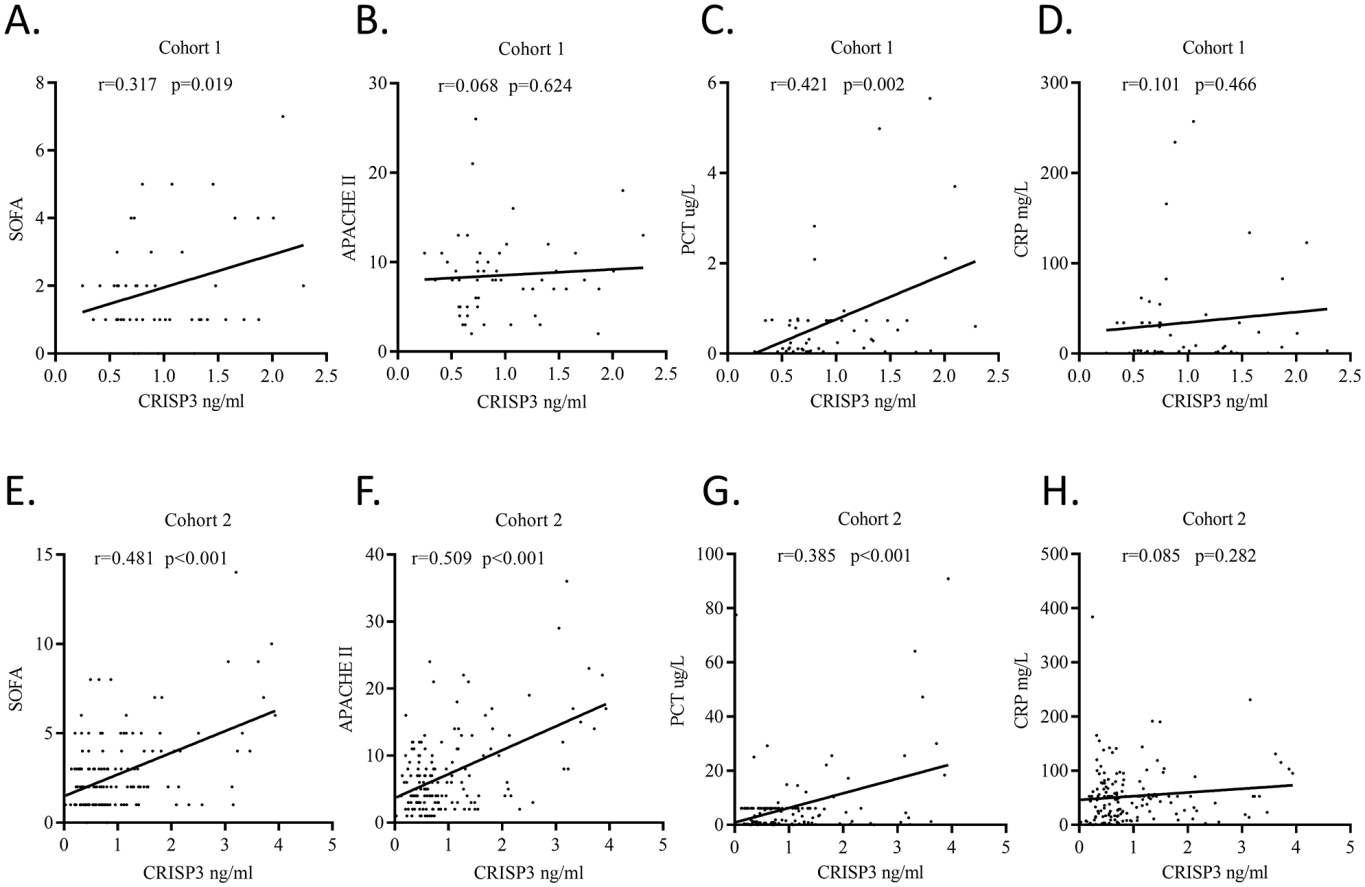
Plasma CRISP3 concentrations were associated with disease severity and inflammatory response of patients. **(A)** SOFA scores and plasma CRISP3 in 54 trauma patients (r = 0.317, p = 0.019). **(B)** APACHE II scores and plasma CRISP3 in 54 trauma patients (r = 0.068, p = 0.624). **(C)** PCT and plasma CRISP3 in 54 trauma patients (r = 0.421, p = 0.002). **(D)** CRP and plasma CRISP3 in 54 trauma patients (r = 0.101, p = 0.466). **(E)** SOFA scores and plasma CRISP3 in 166 trauma patients (r = 0.481, p < 0.001). **(F)** APACHE II scores and plasma CRISP3 in 166 trauma patients (r = 0.509, p < 0.001). **(G)** PCT and plasma CRISP3 in 166 trauma patients (r = 0.385, p < 0.001). **(H)** CRP and plasma CRISP3 in 166 trauma patients (r = 0.085, p = 0.282). CRISP3, cysteine-rich secretory protein 3; SOFA, Sequential Organ Failure Assessment; APACHE, Acute Physiology and Chronic Health Evaluation; PCT, procalcitonin; CRP, C-reactive protein.

We detected plasma CRISP3 values in 54 trauma patients. We compared the CRISP3 plasma concentrations between sepsis and non-sepsis patients, and the results revealed that CRISP3 concentrations were greatly elevated in sepsis patients (1.305 ± 0.535 ng/mL vs. 0.743 ± 0.270 ng/mL, p < 0.001; **Figure 2A**), which were in line with the results of the meta-analysis. Furthermore, the findings suggested that the concentrations of plasma CRISP3 were significantly associated with the risk of trauma sepsis [OR = 1.003 (1.001–1.005), p = 0.001] (**Supplementary Table S3**). Plasma CRISP3 remained significantly associated with a higher risk of sepsis [OR = 1.004 (1.002–1.006), p < 0.001], adjusted by age, sex, and ISS (**Table 2**). To further indicate the role of plasma CRISP3 in sepsis, we explored the relationships between plasma CRISP3 and SOFA, APACHE II scores, procalcitonin (PCT), and C-reactive protein (CRP). The results indicated that CRISP3 concentrations were positively related to SOFA scores (r = 0.317, p = 0.019, **Figure 3A**) and PCT (r = 0.421, p = 0.002, **Figure 3C**), but not APACHE II scores (r = 0.068, p = 0.624, **Figure 3B**) and CRP (r = 0.101, p = 0.466, **Figure 3D**). The above findings suggested that CRISP3 was positively correlated to the severity of sepsis after trauma and may be a predictive biomarker of sepsis patients after trauma.

**Table 2:** Associations between each biomarker and trauma sepsis in adjusted logistic regression models.

Variables	Internal validation cohort		External validation cohort	
	OR (95% CI)	p*	OR (95% CI)	p*
SOFA	2.886 (1.479–5.631)	0.002	1.920 (1.473–2.502)	<0.001
PCT	2.177 (0.927–5.111)	0.074	1.071 (1.010–1.136)	0.022
CRP	1.006 (0.994–1.018)	0.322	0.998 (0.991–1.005)	0.571
CRISP3	1.004 (1.002–1.006)	<0.001	1.002 (1.001–1.003)	<0.001

SOFA, Sequential Organ Failure Assessment; PCT, procalcitonin; CRP, C-reactive protein; CRISP3, cysteine-rich secretory protein 3.

*Adjusted by age, sex, and Injury Severity Score.

**Figure 4:**
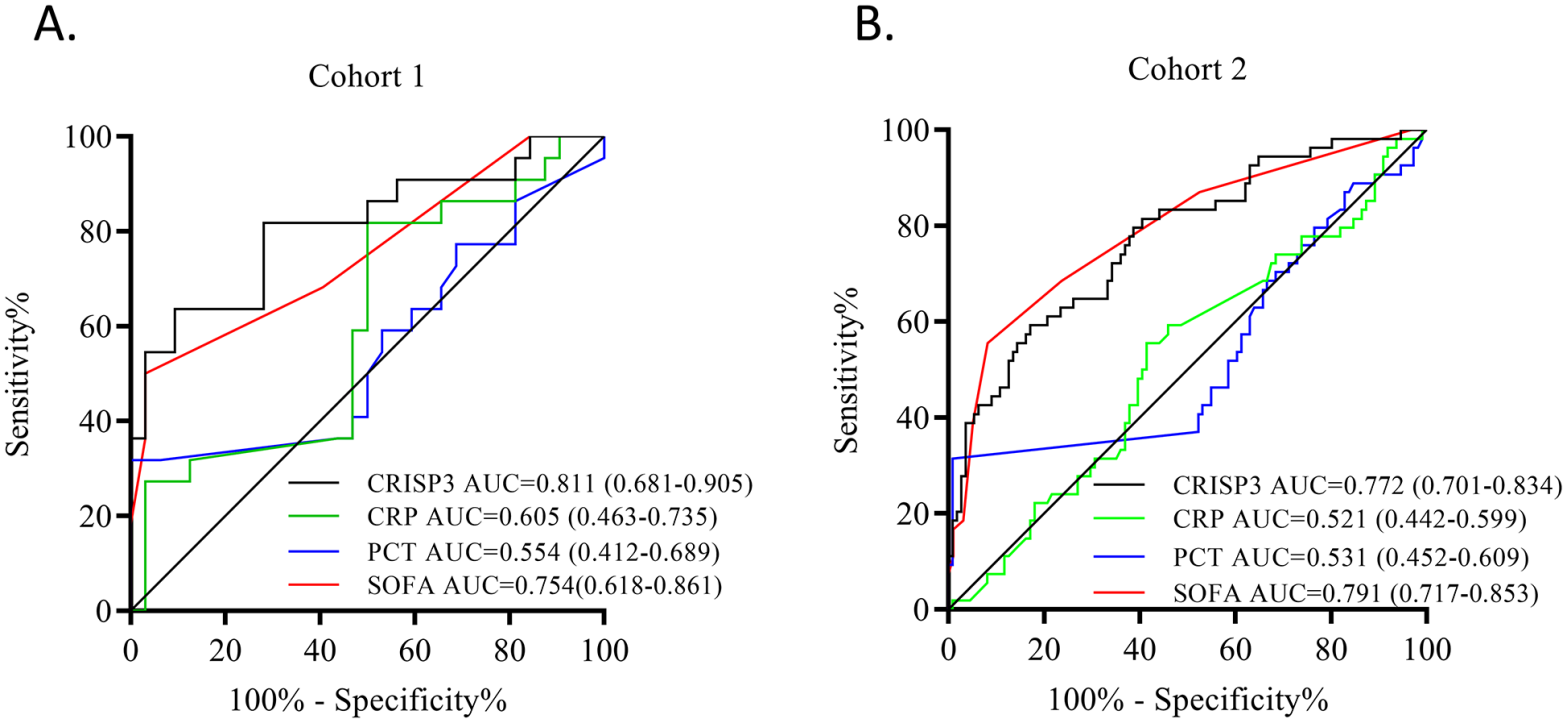
Predictive power of each indicator in sepsis after trauma. **(A)** ROC of CRISP3, CRP, PCT, and SOFA in 54 trauma patients. **(B)** ROC of CRISP3, CRP, PCT, and SOFA in 166 trauma patients. ROC, receiver operating characteristic; CRISP3, cysteine-rich secretory protein 3; CRP, C-reactive protein; PCT, procalcitonin; SOFA, Sequential Organ Failure Assessment.

Subsequently, ROC analysis was adopted to explore the prediction ability of plasma CRISP3. The results revealed that an AUC of 0.811 (0.681–0.905) was obtained for the risk of sepsis after trauma (**Figure 4A**), which had a sensitivity and a specificity of 63.64% and 90.62%, respectively, at the optimal cutoff value of 1.053 ng/mL (**Supplementary Table S4**). Meanwhile, we evaluated the AUCs of CRP, PCT, and SOFA, and the results indicated that the AUCs for sepsis after trauma were 0.605 (0.463–0.735), 0.554 (0.412–0.689), and 0.754 (0.618–0.861), respectively (**Figure 4A**; **Supplementary Table S4**). Compared with those biomarkers and scores, CRISP3 displayed an outperformed AUC (CRP, p = 0.042; PCT, p = 0.004; SOFA, p = 0.576) (**Figure 4A**). Meanwhile, when CRISP3 was combined with SOFA, the AUC was 0.905 (0.794–0.968) (**Supplementary Table S4**), which was superior to that of single CRISP3 or SOFA.

### The validation of plasma CRISP3 for the prediction of sepsis after trauma

To further verify the predictive value of plasma CRISP3 for sepsis after injury, another 166 trauma patients were recruited from a different medical institute. Consistent with the previous findings, sepsis patients had significantly higher plasma CRISP3 compared with non-sepsis patients (1.542 ± 1.075 ng/mL vs. 0.696 ± 0.509 ng/mL, p < 0.001) (**Figure 2B**). Plasma CRISP3 on admission after injury remained significantly related to the occurrence of sepsis [OR = 1.002 (1.001–1.003), p < 0.001] (**Table 2**). Meanwhile, an AUC [0.772 (0.701–0.834)] of plasma CRISP3 was obtained for the risk of sepsis after trauma (**Figure 4B**, **Supplementary Table S5**). The sensitivity and specificity were 59.26% and 82.88%, respectively, at a cutoff value of 1.070 ng/mL in the 166 trauma patients (**Supplementary Table S5**). Meanwhile, plasma CRISP3 was positively associated with SOFA (r = 0.481, p < 0.001, **Figure 3E**), APACHE II scores (r = 0.509, p < 0.001, **Figure 3F**), and PCT (r = 0.385, p < 0.001, **Figure 3G**), but not CRP (r = 0.085, p = 0.282, **Figure 3H**). Furthermore, plasma CRISP3 displayed a better predictive value compared with CRP [AUC = 0.521 (0.442–0.599), p = 0.0004] and PCT [AUC = 0.531 (0.452–0.609), p = 0.0002] but not SOFA [AUC = 0.791 (0.717–0.853), p = 0.5606] (**Figure 4B** and **Supplementary Table S5**). Moreover, the addition of SOFA to CRISP3 improved the predictive accuracy to [0.841 (0.772–0.895)] (**Supplementary Table S5**).

## Discussion

In the present research, we utilized publicly available datasets and two cohorts to systematically explore the association between CRISP3 and sepsis. Through analysis of 23 microarray datasets, we observed an increased expression of CRISP3 in sepsis patients. Additionally, we used two retrospective cohorts to verify the correlations between plasma CRISP3 and sepsis following injury. These findings suggest that increased CRISP3 may serve as a biomarker to identify patients at high risk of sepsis.

With the development of research, various studies have suggested that CRISP3 may be involved in some pathologic processes, such as dengue (12), prostate cancer (42), and breast cancer (43). Dysregulated CRISP3 was related to the undesirable prognosis of several cancers, and CRISP3 was determined as a therapeutic target for those patients (15–17, 44, 45). To explore the expression of CRISP3 in sepsis, we performed a comprehensive analysis based on the expression values from the ArrayExpress and GEO databases. The results of the meta-analysis suggested that the significant upregulation of CRISP3 in sepsis was observed across all datasets. In contrast, there was no significant difference between the sepsis survival and dead patients, which indicated that CRISP3 did not affect the prognosis of sepsis patients. To further determine the upregulation of CRISP3 concentrations in sepsis, we performed two retrospective studies that included 54 and 166 trauma patients. Those results demonstrated that plasma CRISP3 was elevated in the sepsis patients in both cohorts, which was consistent with the meta-analysis results. Thus, all of those results suggested that CRISP3 concentrations could serve as a potential biomarker to predict the risk of post-injury sepsis.

To our knowledge, no reports have described the function of CRISP3 in sepsis. Cumulative reports suggested that CRISP3 participated in innate immunity, defense response, and chronic inflammation (9, 18, 46). Meanwhile, our results indicated that CRISP3 was significantly correlated with PCT, a well-established indicator of inflammation. This further suggests that CRISP3 may contribute to innate immunity. Previous studies have demonstrated that CRISP3 was localized and expressed primarily in neutrophils and thymocytes (47). As the most abundant white blood cells, neutrophils are the first line of defense against invading pathogens; thus, we speculated that CRISP3 may affect the spread of pathogens in sepsis by influencing neutrophil activation and degranulation process. However, the mechanisms of how CRISP3 affects the development of sepsis remain still unclear. A study by Pathak et al. (48) revealed that values of CRISP3 were negatively associated with the expression of annexin A1 (ANXA1). As an anti-inflammatory molecule, ANXA1 was involved in the sepsis process (49, 50). Meanwhile, R1 B-glycoprotein (A1BG), a member of the immunoglobulin superfamily, was identified to bind with CRISP3 in plasma or serum by mass spectrometry (46, 51). Thus, we proposed that CRISP3 may contribute to the development of sepsis by affecting the expression or function of ANXA1 or A1BG. This incomplete understanding required us to investigate the underlying mechanisms of how CRISP3 affects the development of sepsis.

Sepsis biomarkers could direct physicians to prognosticate patient risk and initiate individualized therapy (52, 53). Despite the growing number of potential sepsis biomarkers that were identified, PCT and CRP were the most frequently explored (52, 54). However, the prediction abilities of CRP and PCT for sepsis after trauma were ambiguous (53, 55). In the current study, we demonstrated that the CRP and PCT on hospital admission were weakly associated with the incidence of sepsis after trauma, which was consistent with previous studies (53, 55, 56). Our results found and validated that CRISP3 strongly correlates with the risk of sepsis and that elevated plasma CRISP3 on hospital admission could predict the incidence of sepsis. Compared with CRP and PCT, CRISP3 had an outperformed ability to discriminate sepsis from non-sepsis individuals. Therefore, we suggest that CRISP3 may guide clinicians in the early management of sepsis patients to improve outcomes.

Currently, some inherent limitations need to be noted. First, our retrospective study indicated and verified that CRISP3 increased in trauma sepsis, but the meta-analysis did not include trauma cohorts. These factors may limit the generalizability to other populations. Second, we only found plasma CRISP3 was correlated with the incidence of sepsis. However, how CRISP3 affects the development of sepsis remains unclear. Further studies are warranted to explore the underlying mechanisms among CRISP3 and sepsis. Third, heterogeneity through meta-regression was observed, and the source of heterogeneity should still be considered carefully, including different platforms, methods, and ethnic groups. Finally, the meta-analysis suggested that CRISP3 may not influence the prognosis of sepsis patients; thus, we did not explore the relationship between plasma CRISP3 and the outcomes of sepsis patients.

## Conclusion

Generally, our study determined and verified that CRISP3 was significantly elevated in sepsis patients. More importantly, increased CRISP3 may serve as a latent biomarker to predict the incidence of sepsis. Those findings illustrated a novel target to explore the pathogenesis in sepsis, while more underlying experiments are required to discover the mechanisms of CRISP3 in sepsis.

## Data Availability

The datasets presented in this study can be found in online repositories. The names of the repository/repositories and accession number(s) can be found in the article/**Supplementary Material**.

## Supplementary material

Supplementary Figure 1 Meta-analysis of CRISP3 for the prognosis of sepsis. Forest plot of CRISP3 expression in sepsis-dead and sepsis-survival patients. (Sepsis-dead: n=777; Sepsis-survival: n=1156; SMD=-0.04(-0.16-0.07), p=0.46; *I^2^ =* 29%, p=0.15).

Supplementary Figure 2 Funnel plots of CRISP3 in sepsis to assess publication bias. **(A)** About CRISP3 and the risk of sepsis; **(B)** About CRISP3 and the prognosis of sepsis.
