# Supplementary figures and images for: Upregulation of CRISP3 and its clinical values in adult sepsis: a comprehensive analysis based on microarrays and a two-retrospective-cohort study
Source: Front Immunol. 2024 Nov 18;15:1492538. doi: 10.3389/fimmu.2024.1492538 (PMC11609069; doi:10.3389/fimmu.2024.1492538)

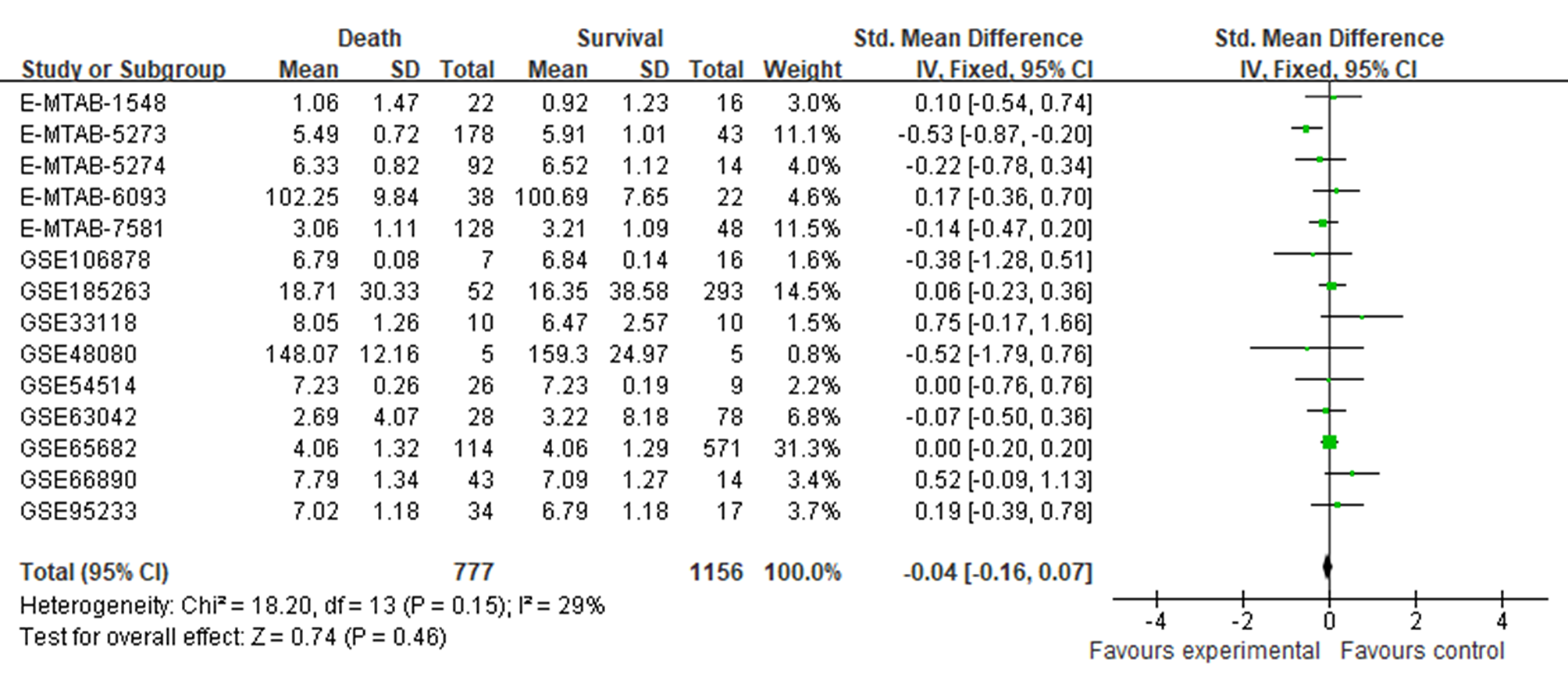

Supplement: Supplementary Figure 1 — Meta-analysis of CRISP3 for the prognosis of sepsis. Forest plot of CRISP3 expression in sepsis-dead and sepsis-survival patients. (Sepsis-dead: n=777; Sepsis-survival: n=1156; SMD=-0.04(-0.16-0.07), p=0.46; I2 = 29%, p=0.15). [file Image1.tif]

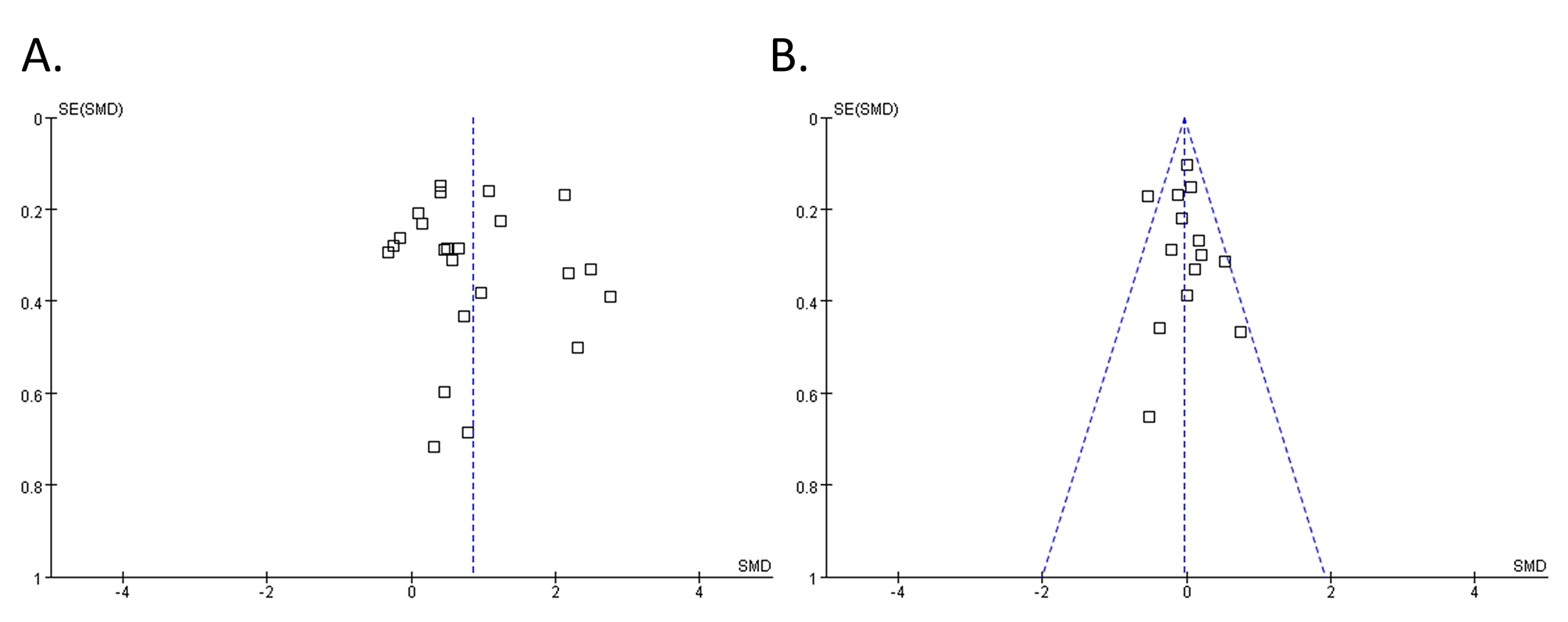

Supplement: Supplementary Figure 2 — Funnel plots of CRISP3 in sepsis to assess publication bias. (A) About CRISP3 and the risk of sepsis; (B) About CRISP3 and the prognosis of sepsis. [file Image2.tif]
