# Supplementary material for: Upregulation of CRISP3 and its clinical values in adult sepsis: a comprehensive analysis based on microarrays and a two-retrospective-cohort study
Source: Front Immunol. 2024 Nov 18;15:1492538. doi: 10.3389/fimmu.2024.1492538 (PMC11609069; doi:10.3389/fimmu.2024.1492538)
Supplement: Supplementary file 3 [file Table1.docx]

**Table S1** the basic characteristics of 23 datasets included in the meta-analysis for the development of sepsis

| **ID** | **Country** | **Year** | **Sepsis** | | | **Control** | | |
| --- | --- | --- | --- | --- | --- | --- | --- | --- |
|  |  |  | **Mean** | **SD** | **n** | **Mean** | **SD** | **n** |
| E-MTAB-1548 | Spain | 2014 | 0.63 | 1.17 | 80 | -0.09 | 0.63 | 15 |
| GSE123729 | Germany | 2019 | 8.08 | 2.00 | 15 | 6.58 | 0.90 | 16 |
| GSE13015 | USA | 2009 | 40.27 | 64.56 | 48 | 10.86 | 2.99 | 3 |
| GSE131761 | Spain | 2020 | 6.92 | 0.13 | 81 | 6.86 | 0.05 | 15 |
| GSE134347 | Malta | 2021 | 6.40 | 1.38 | 156 | 3.99 | 0.32 | 83 |
| GSE185263 | Canada | 2022 | 16.59 | 37.28 | 348 | 2.71 | 3.18 | 52 |
| GSE232753 | South Korea | 2023 | 1.18 | 1.6 | 20 | 0.15 | 0.1 | 8 |
| GSE241238 | China | 2024 | 0.22 | 0.11 | 4 | 0.18 | 0.11 | 4 |
| GSE28750 | Australia | 2011 | 6.56 | 2.35 | 10 | 3.31 | 0.43 | 20 |
| GSE32707 | USA | 2012 | 84.21 | 41.75 | 48 | 91.16 | 37.47 | 21 |
| GSE33118 | France | 2017 | 7.26 | 2.13 | 20 | 3.76 | 1.27 | 42 |
| GSE33341 | USA | 2013 | 5.60 | 1.68 | 51 | 4.00 | 0.52 | 43 |
| GSE40012 | Australia | 2012 | 108.78 | 79.47 | 27 | 72.94 | 7.06 | 18 |
| GSE48080 | Brazil | 2014 | 153.69 | 19.44 | 10 | 138.24 | 13.38 | 3 |
| GSE54514 | Australia | 2014 | 7.23 | 0.21 | 35 | 7.31 | 0.28 | 18 |
| GSE57065 | France | 2014 | 7.78 | 1.72 | 28 | 3.63 | 1.18 | 25 |
| GSE63042 | USA | 2015 | 3.08 | 7.31 | 106 | 2.03 | 4.45 | 23 |
| GSE63990 | USA | 2016 | 6.08 | 1.90 | 70 | 5.48 | 1.09 | 88 |
| GSE6535 | Australia | 2007 | 0.11 | 1.05 | 55 | 0.37 | 0.91 | 17 |
| GSE65682 | Malta | 2015 | 4.06 | 1.30 | 685 | 2.70 | 0.38 | 42 |
| GSE74224 | USA | 2015 | 7.35 | 1.45 | 71 | 7.23 | 1.17 | 34 |
| GSE95233 | France | 2017 | 6.87 | 1.18 | 51 | 4.16 | 0.80 | 22 |
| GSE9960 | Australia | 2009 | 9.16 | 1.93 | 54 | 8.33 | 1.54 | 16 |

**Table S2** The basic characteristics of 14 datasets included in the meta-analysis for the prognosis of sepsis

| **ID** | **Country** | **Year** | **Sepsis-dead** | | | **Sepsis-survival** | | |
| --- | --- | --- | --- | --- | --- | --- | --- | --- |
|  |  |  | **Mean** | **SD** | **n** | **Mean** | **SD** | **n** |
| E-MTAB-1548 | Spain | 2014 | 1.06 | 1.47 | 22 | 0.92 | 1.23 | 16 |
| E-MTAB-5273 | UK | 2017 | 5.49 | 0.72 | 178 | 5.91 | 1.01 | 43 |
| E-MTAB-5274 | UK | 2017 | 6.33 | 0.82 | 92 | 6.52 | 1.12 | 14 |
| E-MTAB-6093 | Thailand | 2017 | 102.25 | 9.84 | 38 | 100.69 | 7.65 | 22 |
| E-MTAB-7581 | UK | 2018 | 3.06 | 1.11 | 128 | 3.21 | 1.09 | 48 |
| GSE106878 | Germany | 2019 | 6.79 | 0.08 | 7 | 6.84 | 0.14 | 16 |
| GSE185263 | Canada | 2023 | 18.71 | 30.33 | 52 | 16.35 | 38.58 | 293 |
| GSE33118 | France | 2017 | 8.05 | 1.26 | 10 | 6.47 | 2.57 | 10 |
| GSE48080 | Brazil | 2014 | 148.07 | 12.16 | 5 | 159.30 | 24.97 | 5 |
| GSE54514 | Australia | 2014 | 7.23 | 0.26 | 26 | 7.23 | 0.19 | 9 |
| GSE63042 | USA | 2015 | 2.69 | 4.07 | 28 | 3.22 | 8.18 | 78 |
| GSE65682 | Malta | 2015 | 4.06 | 1.32 | 114 | 4.06 | 1.29 | 571 |
| GSE66890 | USA | 2015 | 7.79 | 1.34 | 43 | 7.09 | 1.27 | 14 |
| GSE95233 | France | 2017 | 7.02 | 1.18 | 34 | 6.79 | 1.18 | 17 |

**Table S3.** Associations between each variable and traumatic sepsis in univariate analysis

| Variables | **Validation cohort 1** | |  | | **Validation cohort 2** | |
| --- | --- | --- | --- | --- | --- | --- |
|  | OR (95% CI) | P | |  | OR (95% CI) | P |
| CRP | 1.007(0.996-1.018) | 0.211 | |  | 0.998(0.991-1.005) | 0.06 |
| PCT | 2.314 (0.984-5.442) | 0.055 | |  | 1.076(1.020-1.135) | 0.007 |
| SOFA | 2.614(1.426-4.790) | 0.002 | |  | 1.938(1.504-2.497) | <0.001 |
| CRISP3 | 1.003(1.001-1.005) | 0.001 | |  | 1.002(1.001-1.003) | <0.001 |

**Table S4.** Predictive probability of single predictor in validation cohort 1.

| **Variables** | **Validation cohort 1** | | |  |
| --- | --- | --- | --- | --- |
|  | **AUC** | **Sensitivity** | **Specificity** | **Cut-off** |
| CRP | 0.605 (0.463-0.735) | 81.82% | 50.00% | 3.00 |
| PCT | 0.554 (0.412-0.689) | 31.82% | 100.00% | 0.318 |
| SOFA | 0.754(0.618-0.861) | 50.00% | 96.87% | 2.00 |
| CRISP3 | 0.811 (0.681-0.905) | 63.64% | 90.62% | 1.053 |
| CRP+ CRISP3 | 0.822(0.694-0.913) | 72.73% | 97.50% | 0.398 |
| PCT+ CRISP3 | 0.818(0.690-0.910) | 72.73% | 87.50% | 0.419 |
| SOFA+ CRISP3 | 0.905(0.794-0.968) | 86.36% | 87.50% | 0.275 |

AUC: area under curve.

**Table S5.** Predictive probability of single predictor in validation cohort 2.

| **Variables** | **Validation cohort 2** | | |  |
| --- | --- | --- | --- | --- |
|  | **AUC** | **Sensitivity** | **Specificity** | **Cut-off** |
| CRP | 0.521 (0.442-0.599) | 55.56% | 58.56% | 43.00 |
| PCT | 0.531 (0.452-0.609) | 31.48% | 99.10% | 6.11 |
| SOFA | 0.791 (0.717-0.853) | 55.56% | 91.75% | 3.00 |
| CRISP3 | 0.772 (0.701-0.834) | 59.26% | 82.88% | 1.070 |
| CRP+ CRISP3 | 0.777 (0.706-0.838) | 66.67% | 79.28% | 0.298 |
| PCT+ CRISP3 | 0.759 (0.687-0.822) | 59.26% | 84.68% | 0.36 |
| SOFA+ CRISP3 | 0.841 (0.772-0.895) | 83.33% | 78.35% | 0.307 |

AUC: area under curve.
